# Online forum users’ views and experiences of managing irritable bowel syndrome: a qualitative analysis of discussion content

**DOI:** 10.3399/bjgpopen20X101084

**Published:** 2020-10-14

**Authors:** Emma Teasdale, Hannah Clarke, Nick Chen, Hazel Everitt

**Affiliations:** 1 Primary Care, Population Sciences and Medical Education, Faculty of Medicine, University of Southampton, Southampton, UK

**Keywords:** irritable bowel syndrome, self-management, qualitative research, discussion forum, online support group, general practice, primary healthcare

## Abstract

**Background:**

Irritable bowel syndrome (IBS) is common and often negatively affects quality of life. Patients frequently perceive medical interventions as inadequate and seek support from other sources, including online discussion forums.

**Aim:**

To explore online discussion forum topics posted by people with IBS.

**Design & setting:**

A qualitative study exploring three UK-based online discussion forums.

**Method:**

A scoping review identified UK-based discussion forums with high activity and frequent use, which did not require a password/registration to view posts (two IBS-specific and one general health forum). Internal search functions were used to identify and export relevant discussion threads relating to managing IBS. Inductive thematic analysis of exported discussions was undertaken.

**Results:**

Analysis identified two main overarching themes from 122 relevant discussion threads: 1) sharing information and practical advice about lifestyle changes; and 2) receiving emotional support. The most prevalent topics were lifestyle changes, including diet, using oral preparations (for example, supplements or probiotics), and physical activity. Dietary changes were usually considered positive, and most hopeful for potentially alleviating symptoms. Emotional support was also regularly offered with expressions of empathy, kindness, and gratitude, and a sense of users feeling less alone. Some discussions raised concern around potentially inappropriate symptomatic reassurance, and negative or conflicting advice.

**Conclusion:**

Online forums seem, generally, to be a positive experience for those posting, but include potential risks of misinformation. Most posts focus on symptomatic relief through lifestyle change and/or emotional support. Clinicians could gain a better understanding of patients’ ideas, concerns, and expectations of IBS diagnosis and management by asking about patient-acquired online forum information.

## How this fits in

In the non-medical setting of online discussion forums, users predominately shared information and advice around making lifestyles changes (dietary changes and physical activity), and use of oral preparations to help manage IBS symptoms. Dietary changes were considered most hopeful for potentially alleviating symptoms. Online forums seemed, generally, to be a positive experience for those posting, but included potential risks with some discussions raising safety issues, including potentially inappropriate symptomatic reassurance, and examples of negative and conflicting advice. Forums may influence patient expectations of IBS diagnosis and management. GPs asking about online forum use would help them manage these expectations and support self-management.

## Introduction

IBS is a common gastrointestinal disorder that affects 10% to 20% of the global population.^1^ IBS is characterised by abdominal pain, bloating, and altered bowel habit, and can have a significant impact people’s quality of life, social functioning, and work productivity.^2^ Costs for treating patients with IBS are considerable, with NHS UK estimates of £45.6 million to £200 million annually.^3^ National Institute for Health and Care Excellence (NICE) guidance recommends that treatment should focus on a positive diagnosis of IBS, reassurance, and lifestyle advice, including diet and drug treatments.^4^ Despite this, many patients experience ongoing symptoms.

Previous qualitative research has found that patients feel that doctors dismiss or undervalue IBS symptoms, show a lack of empathy during IBS-related consultations, and provide insufficient information about IBS and its management.^5–8^ The diagnostic process is seen as confusing and patients are frequently frustrated by prolonged searches for effective treatments, and this can reduce their trust in doctors and the NHS.^9,10^ In this environment, many people with IBS seek alternative sources of advice and support.

Online platforms are increasingly used to connect with others who have similar health issues.^11^ However, little is known about the discussions taking place online regarding IBS, with few previously published articles in this area.^12,13^ Previous qualitative studies exploring IBS discussions in the online world have focused on social support exchange. Further in-depth exploration of IBS online discussions would enable greater understanding of people’s broader experiences of living with IBS, for example, discussions around symptom management. Online discussions forums can provide a space for users to openly discuss their views and experiences in a natural way without medical input or researcher bias, thus providing qualitative researchers with a rich source of primary, naturalistic data about users’ perspectives and experiences of a particular health issue.^14,15^ Qualitative research exploring the potential impacts of using online forums suggests that online interactions can impact positively and negatively on people’s health, particularly in terms of finding information, feeling supported, changing behaviour based on others’ experiences, and navigating health services.^16^

Awareness of what patients discuss in online discussions can provide clinicians with valuable insight into the perspectives, expectations, and experiences of people managing IBS outside of the clinical setting. This can, in turn, help to identify ways to improve IBS-related consultations. The aim of this study was to explore the content of online discussions about managing IBS.

## Method

This qualitative study sought to systematically explore discussions about managing IBS on three UK-based online discussion forums.

### Data retrieval

In December 2016, a Google search of online resources for people with IBS was conducted by one author (NC) to identify relevant online discussion forums. This scoping review highlighted 12 potential online forums. Each forum was reviewed against inclusion criteria including registration requirements, location, level of recent activity, and number of members, threads, and posts. Online forums that were password protected, not regularly active, and not UK-based were excluded. Three online discussion forums (two IBS specific and one general health forum) met the inclusion criteria. These forums were identified by the research team (NC, medical student; HE, academic GP; ET, qualitative research fellow; and HC, GP registrar) as the largest and most consistently active open access forums based in the UK. All used English language.

Collectively, the forums comprised over 800 000 posts relating to IBS from UK-based IBS charity and general health websites. For anonymity reasons, data on participants was limited but it is likely that they were mostly UK-based adults with IBS or with a relative with IBS. NC familiarised himself with these forums and devised a separate search strategy per forum using internal search functions or topic categorisations to identify discussion threads on managing IBS.

To enable systematic representation of the forum discussions, the first 3 pages of search results per forum (10–30 discussion threads per page) from November 2014 to November 2016 were reviewed. Relevant discussions threads were copied into Microsoft Word verbatim, assigned a unique ID, and then exported to qualitative data management software NVivo (version 11) for analysis.

### Data analysis

An inductive thematic analysis^17^ was conducted of the discussions threads. Discussions were read multiple times in order to become familiar with the data, and then relevant posts were independently coded line by line by two authors (NC and HC). Codes were derived inductively from the data and grouped together to produce an initial coding frame, and from this a detailed coding manual was created to allow systematic coding of the data. Codes were discussed regularly with two further authors (HE and ET) to iteratively develop and refine key themes arising from the data, and to ensure a rich and diverse interpretation of the data. Coding stopped once data saturation of the main themes was achieved.

In terms of ensuring the quality and rigour of this study, Lincoln and Guba’s trustworthiness criteria^18^ were adopted, including creating detailed coding manuals, triangulation of researcher perspectives, and negative case analysis. Reﬂexive thinking was also applied throughout the data collection and analysis, and a clear paper trail was maintained.

### Ethical considerations

Ethical considerations regarding the use of online forum messages for the purposes of qualitative data analysis mostly centre around perceived privacy of forum users, and the extent that messages are considered to be in the public domain.^11^ This study follows British Psychological Society guidelines,^19^ which consider online forum messages to be located within the public domain, provided that no forum registration is required by the researcher to view them, which is an approach widely used in other studies.^12,20–23^ Online forums involved in this study are freely available to the public and open access (not password protected). Following the ethics committee’s guidance, and to protect the anonymity of the data, forums have not been identified and data excerpts have been paraphrased, with any identifiable information removed.

## Results

In total, 122 discussion threads relating to managing IBS were identified (25 from the general health forum and 97 from the two IBS specific forums). Analysis of people’s discussions of managing IBS highlighted two main overarching themes: 1) sharing information and practical advice about lifestyle changes, and 2) receiving emotional support. Both are explored in detail below. Quotes are labelled with the discussion thread, participant, and forum ID.

### Key themes

#### 1. Sharing information and practical advice about lifestyle changes

A dominant discussion topic was people’s views and experiences of changing their lifestyle to improve their IBS symptoms. This typically included sharing information and advice about dietary change, using oral preparations (such as supplements or probiotics), and physical activity.

##### Dietary change

Dietary change (instigating and following specific diets, or avoiding and restricting certain foods) was by far the most common topic of discussion within the threads, and although the information and advice was very varied and not always reliable (medically sound), it was generally perceived positively and considered hopeful for potentially alleviating symptoms. Specific diets were frequently mentioned, including ketogenic diets, ‘the white diet’, ‘specific carbohydrate diet’, and ‘paleo’ diets. The most commonly discussed diet seemed to be the fermentable oligosaccharides, disaccharides, monosaccharides, and polyols (FODMAP) diet.^24–26^

*'A couple of weeks ago, I decided to change my diet to eating only raw fruits and vegetables (* *known as the* *"* *Raw Diet* *"* *)* *— not for my IBS, but for my general health … Totally unexpectedly, my IBS symptoms have been virtually non-existent for the past 3 weeks!!!* ***'*** (Discussion 10, participant 1, IBS forum 2)

*'I began the FODMAP diet a few weeks ago and have not had any episodes and have almost been normal going to the toilet just the odd pain so keeping my fingers crossed as I really think I am dying when I have those episodes as it sure as hell isn't normal'* (Discussion 7, participant 7, IBS forum 2)

A common belief highlighted in the forums was that IBS symptoms are triggered by food intolerance and that eliminating certain foods (rather than food groups, as is common in specific diets) will be effective in managing IBS. While highly varied, individual perceived intolerances were reported in the forums, participants commonly discussed intolerances to wheat, lactose, broccoli, garlic, and onions. It is interesting to note that, while no single diet had exclusively positive references within the forums, the FODMAP diet appeared to have a greater rate of approval, and was also often recommended by other forum users as a first-line option for both symptom control and to identify dietary triggers, and help manage such intolerances:

*'If you haven't already you might want to start a food diary and see if removing certain things from your diet helps. For me keeping away from onion and dairy has made a massive difference'* (Discussion 80, participant 2, IBS forum 1)

Another common topic of discussion was around the consumption of certain food stuffs to help alleviate IBS symptoms. The most frequently recommended single food items appeared to be peppermint, fennel, and aloe vera:

*'I have also found fennel seeds helpful. I infuse fennel seeds and make a tea. I also use chamomile flowers and peppermint leaves to make tea. I'm IBS-C* [IBS with constipation] *and the extra liquids combined with the benefits of the tea has helped me enormously. I also drink about four ounces of Aloe Vera juice each day mixed in with a glass of cold water.'* (Discussion 22, participant 5, IBS forum 2)

*'Has anyone tried peppermint oil capsules? These are miles better than fennel seeds. I believe you should take them three times a day, half hour before each meal, so you could take one when you wake up, one at say 12 noon and one at 5. This relaxes the bowel, allowing the gas to pass out easily — such a relief!!!!'* (Discussion 1, participant 21, IBS forum 2)

Although discussions around dietary change appeared to be mostly positive, some forum users expressed negative experiences and a belief that dietary change is ineffective in managing IBS symptoms, or that IBS requires more than just dietary change alone:

*'Not long ago I tried the low FODMAP diet and my symptoms got much worse! My diarrhoea used to happen every few days and bloating was usually tolerable. However, the low FODMAP diet gave me diarrhoea after every meal, even things like plain rice with chicken. I was on it for a couple of months and that's all I could manage.'* (Discussion 56, participant 9, IBS Forum 1)

*'IBS in my experience is in part a reaction to food and partly psychological. Try the FODMAP diet, strictly, for 6 weeks,*

*· Increase* *exercise, preferably daily. Deep breathing to reduce anxiety and listening to relaxing music These things have helped me greatly over the last 4 weeks. Best of luck'* (Discussion 8, participant 7, IBS forum 2)

##### Using non-dietary oral preparations (food supplements)

Taking food supplements alongside foods or meals, or simply to complement the daily diet was another topic of discussion highlighted in the forums. Participants commonly discuss their experiences with probiotics and vitamin supplements (calcium for diarrhoea and magnesium for constipation). A common belief expressed in these discussions was that probiotics are effective in IBS symptom resolution because IBS is caused by abnormal growth of bacteria (dysbiosis) within the gut. Typical discussions involved recommending formulations of probiotics, and advice was often sought and given regarding brands and the number of bacteria contained:

*'I have had success by supplementing with PROBIOTICS. I will never take anything less than 100 billion and less than 10 strains. The one I’m currently taking is over 150 billion and has over 40 strains. My symptoms have improved significantly.'* (Discussion 67, participant 1, IBS forum 1)

*'I began taking magnesium tablets last week, which has really helped the constipation. I have been constantly constipated for a long time but since starting this I've had a bowel movement nearly every day. I hope this lasts, I haven't had stomach pains really either since starting this.'* (Discussion 21, participant 8, IBS forum 2)

##### Physical activity

Physical activity and relaxation techniques or habits (including physically relaxing the body and its muscles, or less literally in the sense of stress or anxiety relief) were also a popular topic of discussion among users across all three forums, with stated benefits including helping people to relax and enjoy themselves, as well as more directly alleviating their IBS symptoms. Common activities recommended within forums including walking, running, swimming, and yoga:

*'I suffer from IBS-D* [IBS with diarrhoea]*, which is debilitating when it happens. It’s been triggered by my lifestyle and stresses and anxiety that has built up. I am trying to combat this with exercise, relaxation and stress relief'* (Discussion 10, participant 8, IBS forum 2)

*'I've been going to the swimming pool which helped immensely!. I have started some classes there with other people who are pretty close to me, but I don't mind at all because I feel very safe inside the water... I feel like they can't smell if I fart... I'm in the back of the class though. But it feels good. I do exercise and I'm around people without feeling bad!* ***'*** (Discussion 43, participant 1, IBS forum 1)

Some users expressed an inability to exercise and this was typically accompanied by an explanation of how it would exacerbate their pain or increase the sensation of discomfort. However, they often spoke about exercise in a manner that would not dissuade others from trying it out:

*'I would love to do more exercise but for me it's not possible. Yes, it definitely makes the pain worse with me.'* (Discussion 36, participant 4, IBS forum 1)

However, while there appeared to be a general feeling of positivity towards increasing physical activity to improve IBS symptoms, there was not the same optimism expressed about dietary change in terms of effective symptom management and possibly providing a ‘cure’.

#### 2. Receiving emotional support

A dominant perceived effect of using online forums for managing IBS symptoms appeared to be gaining emotional support and feeling less alone. By sharing experiences of embarrassing symptoms and physical health concerns, as well as the impact on daily life and social functioning (for example, concern about access to toilets, eating out, or dealing with abdominal pain and discomfort), forum users received confirmation that others were experiencing similar symptoms and emotional reactions that provided reassurance, comfort, and a sense of solidarity or community:

*'I've now had the "*change*" (as I call it) from IBS-C to D ... I was fine then suddenly I had to go!! It looked like melted chocolate! Pain was terrible! I felt queasy before and whilst doing the business! I just feel so alone with these symptoms! It's ruining day and weeks for me and I just want it to end!!! Does anyone else suffer anything like this? Am I right to feel down and depressed about it?!?!'* (Discussion 7 , participant 1, IBS forum 2)

*'I get that every so often as well. Usually after a few days of IBS-c, my digestive system gives up and implodes at least that is what it feels like. I get pain, nausea, cold sweats and feel dizzy during this IBS-d session, and I feel horrible until it's all over and out of me. It used to happen once a month or so, but lately it's happening less. So no, you're not alone, trust me.'* (Discussion 7, participant 2, IBS forum 2)

*'At last someone who understands!! The sweats are the worst ... That in addition to feeling sick is just unbearable! I honestly felt I was alone. The next day I'm back to C but this as you say happens once every so often but when it does it takes over!'* (Discussion 7, participant 4, IBS forum 2)

There was also a sense of gratitude about receiving emotional support evident in the data. Forum users expressed their appreciation of this online support, particularly in terms of acknowledgement of shared experiences and feeling understood, which they felt was lacking in their offline communication (for example, with health professionals or significant others):

*'Thank you again for all the support, posting here has been the best decision of my life. I feel so happy that everyone understands how I am feeling instead of thinking me as weird or just thinking that I am overreacting to a small fault in the functioning of my bowel health.'* (Discussion 62 , participant 9, IBS forum 1)

*'I know how you feel! In the past when I tried to get support from other IBS sufferers, some of my friends came up with daft responses like* *"* *Just go see a doctor* *"* *which made me feel really frustrated. Sometimes it takes a bit of education as they don't know how it feels unless they have this. I don't blame them unless they say unhelpful things which are common sense, especially when I’ve been to more than two doctors already'* (Discussion 19, participant 3, IBS forum 2)

While most of the information and advice shared in the forums was supportive and appeared to be in line with good GP advice and NICE guidelines, there were examples of speculative or conflicting advice. Some users spoke about significant changes in their IBS symptoms (for example, rapid weight loss, rectal bleeding), and while responders often suggested they seek immediate further advice from their GPs, this was not always the case. There were also examples of negative and conflicting advice that other users perceived as fear-mongering:

*'I try to monitor what I eat so I don't cause flare ups. I notice my weight hasn’t changed. In 2014 i was 90 pounds, then in 2015 I dropped to 88, 83 and now I am at 86 pounds. Is this normal? Are you guys able to put on weight easily?'* (Discussion 11, participant 1, IBS forum 1)

*‘I have IBS-C and I am experiencing weight loss as well, but it is from a fungal issue and possibly SIBO* [small intestinal bacterial overgrowth]*. The bacteria and fungus eat all your nutrients. The candida develops because of bacteria imbalance. Can I ask what diet are you on?'* (Discussion 11, participant 2, IBS forum 1)

*'Aloe Vera is a wonderful short-term gut remedy, but we are warned against long- term use because it is a laxative & will turn the intestinal walls form a healthy pink to black, and possibly increase the risk of colonic cancer. Even after stopping, the colon may only partially return to normal because of permanent drug-induced damage … Will you be taking Aloe in any form now knowing of these risks?? I CERTAINLY WILL NOT.’* (Discussion 95, participant 15, IBS forum 2)

*‘G’s Aloe doesn't have these properties. It tastes like clean water and is tremendously effective. Stop fear mongering”* (Discussion 95, participant 16, IBS forum 2)

## Discussion

### Summary

The analysis of online discussion forum topics posted by people with IBS revealed two themes: sharing information and practical advice about lifestyle changes, and receiving emotional support. Online forums seem generally a positive experience for those posting but include potential risks, with some discussions raising concerns around potentially inappropriate symptomatic reassurance, and negative or conflicting advice.

### Strengths and limitations

Few research studies have explored online discussion forum topics posted by people with IBS, and these were published quite some time ago.^12,13^ To the authors' knowledge, this study was the first to explore freely accessible, predominantly UK-based forums. The aim was to capture a representative sample of forum posts by undertaking a scoping review to identify active UK-based discussion forums that did not require a password or registration to view posts, and by including a range of active forums (two IBS specific forums and one general health forum). The study followed the British Psychological Society guidelines^19^ to ensure ethical and rigorous research procedures, and had a multidisciplinary team including clinicians and health psychologists, who brought a range of perspectives to data analysis and interpretation.

Limitations include that all forum research had limited demographic information on the people posting, so the authors did not know the age, sex/gender, ethnicity, or disease severity of those contributing data, compared to those who seek GP advice for IBS. As data were collected retrospectively, the analysis was limited to the data available as it was not feasible to ask users to clarify or expand on their posts. While online platforms are increasingly being used to communicate with others who have similar health issues, the prevalence of online forum usage by people with IBS is not known. One study that explored the pattern of engagement with forums and Facebook groups for people with inflammatory bowel disease (IBD) revealed sociodemographic differences in the type of support accessed, with Facebook group members more likely to be younger, more educated, and single.^27^ Forum users may have had different experiences to people with IBS who do not access forums for advice, so this study's findings cannot be generalised outside this specific context. Nevertheless, the consistency of these findings with similar research suggests these are important findings to share with clinicians.

### Comparison with existing literature

This study's findings are consistent with previous qualitative research on IBS^12,13^ and other gastrointestinal conditions such as IBD,^28,29^ which suggests online forums provide people managing IBS with a useful platform for exchanging both informational and social support. These studies propose that reciprocal online social support helps to build stable and well-connected networks that provide users with a sense of community, and enable communication of informational support around symptom interpretation, illness management, and interactions with health professionals. This study found that through exchanging advice and shared experiences, users felt understood and less alone. Social Learning Theory^30^ suggests that people learn by modelling others’ behaviours. Observing how others cope with and manage a condition via online platforms can help to validate or normalise illness experiences.

Existing qualitative research also found that patients feel that doctors dismiss or undervalue IBS symptoms, show a lack of empathy during IBS-related consultations, and provide insufficient information about IBS and its management.^5–8^ The diagnostic process is seen as confusing, and patients are frequently frustrated by prolonged searches for effective treatments, which can reduce their trust in doctors and the NHS.^9,10^ There was some evidence of this in this study's data but there appeared to be greater focus on the value of the support received online, with more indirect references to dissatisfaction with offline information and advice on IBS.

Qualitative research exploring the potential impacts of using online forums suggests that online interactions can impact positively and negatively on people’s health.^16,28^ This is, again, consistent with this study's findings, which highlighted that using IBS online forums seemed to be generally a positive experience but could include potential risks of misinformation and negative advice.

### Implications for research and practice

Participating in online forums may influence people’s experiences and has implications for understanding of individual’s role in managing their IBS. This study reports useful exploratory work looking at people’s views about managing IBS. However, the analysis was limited to the data available as they were collected retrospectively. Further primary qualitative research that allows for clarification and more in-depth exploration of people’s views and experiences of IBS would be beneficial. Further research that examines the prevalence of online forums use and other online platforms, such social media sites, would also be beneficial.

Previous research has shown that patients with IBS feel that doctors dismiss or undervalue IBS symptoms, show a lack of empathy during IBS-related consultations, and provide insufficient information about IBS and its management. This research reveals the broad range of advice and support IBS patients seek from online forums and highlights the many non-medical ways they try to manage their IBS. Online forums seem generally to be a positive experience for those posting but can include potential risks. Forums may influence patient expectations of IBS diagnosis and management. GPs should ask people with IBS about their use of online forums. This could reveal valuable further insight into patients’ perspectives, expectations, and experiences, and help GPs to support patient self-management.
